# Supplementary material for: End Sequence Analysis Toolkit (ESAT) expands the extractable information from single-cell RNA-seq data
Source: Genome Res. 2016 Oct;26(10):1397–410. doi: 10.1101/gr.207902.116 (PMC5052061; doi:10.1101/gr.207902.116)
Supplement: Supplemental Material [file supp_gr.207902.116_Supp_Materials.tar.gz › ESAT-esat_v0.1/doc/html/class_jama_1_1_matrix.html]

ESAT: Jama.Matrix Class Reference


ESAT


- Main Page
- Related Pages
- Packages
- Classes
- Files

- Class List
- Class Index
- Class Hierarchy
- Class Members

All Classes Namespaces Files Functions Variables Enumerator Pages

- Jama
- Matrix

Public Member Functions |
Static Public Member Functions |
List of all members

Jama.Matrix Class Reference

Inheritance diagram for Jama.Matrix:

[legend]

Collaboration diagram for Jama.Matrix:

[legend]

|  |  |
| --- | --- |
| Public Member Functions | |
|  | Matrix (int m, int n) |
|  | |
|  | Matrix (int m, int n, double s) |
|  | |
| int | getNumRows () |
|  | |
| int | getNumColumns () |
|  | |
|  | Matrix (double[][] A) |
|  | |
|  | Matrix (double[][] A, int m, int n) |
|  | |
|  | Matrix (double vals[], int m) |
|  | |
| Matrix | copy () |
|  | |
| Object | clone () |
|  | |
| double[][] | getArray () |
|  | |
| double[][] | getArrayCopy () |
|  | |
| double[] | getColumnPackedCopy () |
|  | |
| double[] | getRowPackedCopy () |
|  | |
| int | getRowDimension () |
|  | |
| int | getColumnDimension () |
|  | |
| double | get (int i, int j) |
|  | |
| double[] | getRow (int i) |
|  | |
| double[] | getColumn (int j) |
|  | |
| Matrix | getMatrix (int i0, int i1, int j0, int j1) |
|  | |
| Matrix | getMatrix (int[] r, int[] c) |
|  | |
| Matrix | getMatrix (int i0, int i1, int[] c) |
|  | |
| Matrix | getMatrix (int[] r, int j0, int j1) |
|  | |
| void | set (int i, int j, double s) |
|  | |
| void | setMatrix (int i0, int i1, int j0, int j1, Matrix X) |
|  | |
| void | setMatrix (int[] r, int[] c, Matrix X) |
|  | |
| void | setMatrix (int[] r, int j0, int j1, Matrix X) |
|  | |
| void | setMatrix (int i0, int i1, int[] c, Matrix X) |
|  | |
| Matrix | transpose () |
|  | |
| double | norm1 () |
|  | |
| double | norm2 () |
|  | |
| double | normInf () |
|  | |
| double | normF () |
|  | |
| Matrix | uminus () |
|  | |
| Matrix | plus (Matrix B) |
|  | |
| Matrix | plusEquals (Matrix B) |
|  | |
| Matrix | minus (Matrix B) |
|  | |
| Matrix | minusEquals (Matrix B) |
|  | |
| Matrix | arrayTimes (Matrix B) |
|  | |
| Matrix | arrayTimesEquals (Matrix B) |
|  | |
| Matrix | arrayRightDivide (Matrix B) |
|  | |
| Matrix | arrayRightDivideEquals (Matrix B) |
|  | |
| Matrix | arrayLeftDivide (Matrix B) |
|  | |
| Matrix | arrayLeftDivideEquals (Matrix B) |
|  | |
| Matrix | times (double s) |
|  | |
| Matrix | timesEquals (double s) |
|  | |
| Matrix | times (Matrix B) |
|  | |
| LUDecomposition | lu () |
|  | |
| QRDecomposition | qr () |
|  | |
| CholeskyDecomposition | chol () |
|  | |
| SingularValueDecomposition | svd () |
|  | |
| EigenvalueDecomposition | eig () |
|  | |
| Matrix | solve (Matrix B) |
|  | |
| Matrix | solveTranspose (Matrix B) |
|  | |
| Matrix | inverse () |
|  | |
| double | det () |
|  | |
| int | rank () |
|  | |
| double | cond () |
|  | |
| double | trace () |
|  | |
| void | print (int w, int d) |
|  | |
| void | print (PrintWriter output, int w, int d) |
|  | |
| void | print (NumberFormat format, int width) |
|  | |
| void | print (PrintWriter output, NumberFormat format, int width) |
|  | |
| boolean | isSymmetric () |
|  | |
| boolean | isDiagonal () |
|  | |
| void | quantileNormalizeColumns () |
|  | |
| Matrix | sortColumnsKeepingIndex (Matrix index) |
|  | |
| Matrix | sortColumns () |
|  | |
| void | setColumn (int column, double[] vals) |
|  | |
| boolean | setRow (int row, double[] vals) |
|  | |
| boolean | setRow (int row, int[] vals) |
|  | |
| String | toString () |
|  | |
| void | write (String save, double[] observed) throws IOException |
|  | |

|  |  |
| --- | --- |
| Static Public Member Functions | |
| static Matrix | constructWithCopy (double[][] A) |
|  | |
| static Matrix | random (int m, int n) |
|  | |
| static Matrix | identity (int m, int n) |
|  | |
| static Matrix | read (BufferedReader input) throws java.io.IOException |
|  | |

## Detailed Description

Jama = Java Matrix class.

The Java Matrix Class provides the fundamental operations of numerical linear algebra. Various constructors create Matrices from two dimensional arrays of double precision floating point numbers. Various "gets" and "sets" provide access to submatrices and matrix elements. Several methods implement basic matrix arithmetic, including matrix addition and multiplication, matrix norms, and element-by-element array operations. Methods for reading and printing matrices are also included. All the operations in this version of the Matrix Class involve real matrices. Complex matrices may be handled in a future version.

Five fundamental matrix decompositions, which consist of pairs or triples of matrices, permutation vectors, and the like, produce results in five decomposition classes. These decompositions are accessed by the Matrix class to compute solutions of simultaneous linear equations, determinants, inverses and other matrix functions. The five decompositions are:

- Cholesky Decomposition of symmetric, positive definite matrices.
- LU Decomposition of rectangular matrices.
- QR Decomposition of rectangular matrices.
- Singular Value Decomposition of rectangular matrices.
- Eigenvalue Decomposition of both symmetric and nonsymmetric square matrices.

**Example of use:**
:   Solve a linear system A x = b and compute the residual norm, ||b - A x||.

    ```
          double[][] vals = {{1.,2.,3},{4.,5.,6.},{7.,8.,10.}};
          Matrix A = new Matrix(vals);
          Matrix b = Matrix.random(3,1);
          Matrix x = A.solve(b);
          Matrix r = A.times(x).minus(b);
          double rnorm = r.normInf();
    ```

Author
:   The MathWorks, Inc. and the National Institute of Standards and Technology.

Version
:   5 August 1998

## Constructor & Destructor Documentation

|  |  |  |  |
| --- | --- | --- | --- |
| Jama.Matrix.Matrix | ( | int | *m*, |
|  |  | int | *n* |
|  | ) |  |  |

Construct an m-by-n matrix of zeros.

Parameters
:   |  |  |
    | --- | --- |
    | m | Number of rows. |
    | n | Number of colums. |

Here is the caller graph for this function:

|  |  |  |  |
| --- | --- | --- | --- |
| Jama.Matrix.Matrix | ( | int | *m*, |
|  |  | int | *n*, |
|  |  | double | *s* |
|  | ) |  |  |

Construct an m-by-n constant matrix.

Parameters
:   |  |  |
    | --- | --- |
    | m | Number of rows. |
    | n | Number of colums. |
    | s | Fill the matrix with this scalar value. |

|  |  |  |  |  |  |
| --- | --- | --- | --- | --- | --- |
| Jama.Matrix.Matrix | ( | double | *A*[][] | ) |  |

Construct a matrix from a 2-D array.

Parameters
:   |  |  |
    | --- | --- |
    | A | Two-dimensional array of doubles. |

Exceptions
:   |  |  |
    | --- | --- |
    | IllegalArgumentException | All rows must have the same length |

See also
:   constructWithCopy

|  |  |  |  |
| --- | --- | --- | --- |
| Jama.Matrix.Matrix | ( | double | *A*[][], |
|  |  | int | *m*, |
|  |  | int | *n* |
|  | ) |  |  |

Construct a matrix quickly without checking arguments.

Parameters
:   |  |  |
    | --- | --- |
    | A | Two-dimensional array of doubles. |
    | m | Number of rows. |
    | n | Number of colums. |

|  |  |  |  |
| --- | --- | --- | --- |
| Jama.Matrix.Matrix | ( | double | *vals*[], |
|  |  | int | *m* |
|  | ) |  |  |

Construct a matrix from a one-dimensional packed array

Parameters
:   |  |  |
    | --- | --- |
    | vals | One-dimensional array of doubles, packed by columns (ala Fortran). |
    | m | Number of rows. |

Exceptions
:   |  |  |
    | --- | --- |
    | IllegalArgumentException | Array length must be a multiple of m. |

## Member Function Documentation

|  |  |  |  |  |  |
| --- | --- | --- | --- | --- | --- |
| Matrix Jama.Matrix.arrayLeftDivide | ( | Matrix | *B* | ) |  |

Element-by-element left division, C = A.

Parameters
:   |  |  |
    | --- | --- |
    | B | another matrix |

Returns
:   A.

Here is the call graph for this function:

|  |  |  |  |  |  |
| --- | --- | --- | --- | --- | --- |
| Matrix Jama.Matrix.arrayLeftDivideEquals | ( | Matrix | *B* | ) |  |

Element-by-element left division in place, A = A.

Parameters
:   |  |  |
    | --- | --- |
    | B | another matrix |

Returns
:   A.

|  |  |  |  |  |  |
| --- | --- | --- | --- | --- | --- |
| Matrix Jama.Matrix.arrayRightDivide | ( | Matrix | *B* | ) |  |

Element-by-element right division, C = A./B

Parameters
:   |  |  |
    | --- | --- |
    | B | another matrix |

Returns
:   A./B

Here is the call graph for this function:

|  |  |  |  |  |  |
| --- | --- | --- | --- | --- | --- |
| Matrix Jama.Matrix.arrayRightDivideEquals | ( | Matrix | *B* | ) |  |

Element-by-element right division in place, A = A./B

Parameters
:   |  |  |
    | --- | --- |
    | B | another matrix |

Returns
:   A./B

|  |  |  |  |  |  |
| --- | --- | --- | --- | --- | --- |
| Matrix Jama.Matrix.arrayTimes | ( | Matrix | *B* | ) |  |

Element-by-element multiplication, C = A.\*B

Parameters
:   |  |  |
    | --- | --- |
    | B | another matrix |

Returns
:   A.\*B

Here is the call graph for this function:

|  |  |  |  |  |  |
| --- | --- | --- | --- | --- | --- |
| Matrix Jama.Matrix.arrayTimesEquals | ( | Matrix | *B* | ) |  |

Element-by-element multiplication in place, A = A.\*B

Parameters
:   |  |  |
    | --- | --- |
    | B | another matrix |

Returns
:   A.\*B

|  |  |  |  |  |
| --- | --- | --- | --- | --- |
| CholeskyDecomposition Jama.Matrix.chol | ( |  | ) |  |

Cholesky Decomposition

Returns
:   CholeskyDecomposition

See also
:   CholeskyDecomposition

|  |  |  |  |  |
| --- | --- | --- | --- | --- |
| Object Jama.Matrix.clone | ( |  | ) |  |

Clone the Matrix object.

|  |  |  |  |  |
| --- | --- | --- | --- | --- |
| double Jama.Matrix.cond | ( |  | ) |  |

Matrix condition (2 norm)

Returns
:   ratio of largest to smallest singular value.

|  |  |  |  |  |  |
| --- | --- | --- | --- | --- | --- |
| static Matrix Jama.Matrix.constructWithCopy | ( | double | *A*[][] | ) |  |

static

Construct a matrix from a copy of a 2-D array.

Parameters
:   |  |  |
    | --- | --- |
    | A | Two-dimensional array of doubles. |

Exceptions
:   |  |  |
    | --- | --- |
    | IllegalArgumentException | All rows must have the same length |

Here is the call graph for this function:

|  |  |  |  |  |
| --- | --- | --- | --- | --- |
| Matrix Jama.Matrix.copy | ( |  | ) |  |

Make a deep copy of a matrix

Here is the call graph for this function:

Here is the caller graph for this function:

|  |  |  |  |  |
| --- | --- | --- | --- | --- |
| double Jama.Matrix.det | ( |  | ) |  |

Matrix determinant

Returns
:   determinant

|  |  |  |  |  |
| --- | --- | --- | --- | --- |
| EigenvalueDecomposition Jama.Matrix.eig | ( |  | ) |  |

Eigenvalue Decomposition

Returns
:   EigenvalueDecomposition

See also
:   EigenvalueDecomposition

|  |  |  |  |
| --- | --- | --- | --- |
| double Jama.Matrix.get | ( | int | *i*, |
|  |  | int | *j* |
|  | ) |  |  |

Get a single element.

Parameters
:   |  |  |
    | --- | --- |
    | i | Row index. |
    | j | Column index. |

Returns
:   A(i,j)

Exceptions
:   |  |  |
    | --- | --- |
    | ArrayIndexOutOfBoundsException |  |

Here is the caller graph for this function:

|  |  |  |  |  |
| --- | --- | --- | --- | --- |
| double [][] Jama.Matrix.getArray | ( |  | ) |  |

Access the internal two-dimensional array.

Returns
:   Pointer to the two-dimensional array of matrix elements.

|  |  |  |  |  |
| --- | --- | --- | --- | --- |
| double [][] Jama.Matrix.getArrayCopy | ( |  | ) |  |

Copy the internal two-dimensional array.

Returns
:   Two-dimensional array copy of matrix elements.

|  |  |  |  |  |  |
| --- | --- | --- | --- | --- | --- |
| double [] Jama.Matrix.getColumn | ( | int | *j* | ) |  |

Here is the caller graph for this function:

|  |  |  |  |  |
| --- | --- | --- | --- | --- |
| int Jama.Matrix.getColumnDimension | ( |  | ) |  |

Get column dimension.

Returns
:   n, the number of columns.

Here is the caller graph for this function:

|  |  |  |  |  |
| --- | --- | --- | --- | --- |
| double [] Jama.Matrix.getColumnPackedCopy | ( |  | ) |  |

Make a one-dimensional column packed copy of the internal array.

Returns
:   Matrix elements packed in a one-dimensional array by columns.

|  |  |  |  |
| --- | --- | --- | --- |
| Matrix Jama.Matrix.getMatrix | ( | int | *i0*, |
|  |  | int | *i1*, |
|  |  | int | *j0*, |
|  |  | int | *j1* |
|  | ) |  |  |

Get a submatrix.

Parameters
:   |  |  |
    | --- | --- |
    | i0 | Initial row index |
    | i1 | Final row index |
    | j0 | Initial column index |
    | j1 | Final column index |

Returns
:   A(i0:i1,j0:j1)

Exceptions
:   |  |  |
    | --- | --- |
    | ArrayIndexOutOfBoundsException | Submatrix indices |

Here is the call graph for this function:

|  |  |  |  |
| --- | --- | --- | --- |
| Matrix Jama.Matrix.getMatrix | ( | int[] | *r*, |
|  |  | int[] | *c* |
|  | ) |  |  |

Get a submatrix.

Parameters
:   |  |  |
    | --- | --- |
    | r | Array of row indices. |
    | c | Array of column indices. |

Returns
:   A(r(:),c(:))

Exceptions
:   |  |  |
    | --- | --- |
    | ArrayIndexOutOfBoundsException | Submatrix indices |

Here is the call graph for this function:

|  |  |  |  |
| --- | --- | --- | --- |
| Matrix Jama.Matrix.getMatrix | ( | int | *i0*, |
|  |  | int | *i1*, |
|  |  | int[] | *c* |
|  | ) |  |  |

Get a submatrix.

Parameters
:   |  |  |
    | --- | --- |
    | i0 | Initial row index |
    | i1 | Final row index |
    | c | Array of column indices. |

Returns
:   A(i0:i1,c(:))

Exceptions
:   |  |  |
    | --- | --- |
    | ArrayIndexOutOfBoundsException | Submatrix indices |

Here is the call graph for this function:

|  |  |  |  |
| --- | --- | --- | --- |
| Matrix Jama.Matrix.getMatrix | ( | int[] | *r*, |
|  |  | int | *j0*, |
|  |  | int | *j1* |
|  | ) |  |  |

Get a submatrix.

Parameters
:   |  |  |
    | --- | --- |
    | r | Array of row indices. |
    | i0 | Initial column index |
    | i1 | Final column index |

Returns
:   A(r(:),j0:j1)

Exceptions
:   |  |  |
    | --- | --- |
    | ArrayIndexOutOfBoundsException | Submatrix indices |

Here is the call graph for this function:

|  |  |  |  |  |
| --- | --- | --- | --- | --- |
| int Jama.Matrix.getNumColumns | ( |  | ) |  |

|  |  |  |  |  |
| --- | --- | --- | --- | --- |
| int Jama.Matrix.getNumRows | ( |  | ) |  |

Here is the caller graph for this function:

|  |  |  |  |  |  |
| --- | --- | --- | --- | --- | --- |
| double [] Jama.Matrix.getRow | ( | int | *i* | ) |  |

|  |  |  |  |  |
| --- | --- | --- | --- | --- |
| int Jama.Matrix.getRowDimension | ( |  | ) |  |

Get row dimension.

Returns
:   m, the number of rows.

Here is the caller graph for this function:

|  |  |  |  |  |
| --- | --- | --- | --- | --- |
| double [] Jama.Matrix.getRowPackedCopy | ( |  | ) |  |

Make a one-dimensional row packed copy of the internal array.

Returns
:   Matrix elements packed in a one-dimensional array by rows.

|  |  |  |  |
| --- | --- | --- | --- |
| static Matrix Jama.Matrix.identity | ( | int | *m*, |
|  |  | int | *n* |
|  | ) |  |  |

static

Generate identity matrix

Parameters
:   |  |  |
    | --- | --- |
    | m | Number of rows. |
    | n | Number of colums. |

Returns
:   An m-by-n matrix with ones on the diagonal and zeros elsewhere.

Here is the call graph for this function:

Here is the caller graph for this function:

|  |  |  |  |  |
| --- | --- | --- | --- | --- |
| Matrix Jama.Matrix.inverse | ( |  | ) |  |

Matrix inverse or pseudoinverse

Returns
:   inverse(A) if A is square, pseudoinverse otherwise.

Here is the call graph for this function:

|  |  |  |  |  |
| --- | --- | --- | --- | --- |
| boolean Jama.Matrix.isDiagonal | ( |  | ) |  |

|  |  |  |  |  |
| --- | --- | --- | --- | --- |
| boolean Jama.Matrix.isSymmetric | ( |  | ) |  |

|  |  |  |  |  |
| --- | --- | --- | --- | --- |
| LUDecomposition Jama.Matrix.lu | ( |  | ) |  |

LU Decomposition

Returns
:   LUDecomposition

See also
:   LUDecomposition

|  |  |  |  |  |  |
| --- | --- | --- | --- | --- | --- |
| Matrix Jama.Matrix.minus | ( | Matrix | *B* | ) |  |

C = A - B

Parameters
:   |  |  |
    | --- | --- |
    | B | another matrix |

Returns
:   A - B

Here is the call graph for this function:

|  |  |  |  |  |  |
| --- | --- | --- | --- | --- | --- |
| Matrix Jama.Matrix.minusEquals | ( | Matrix | *B* | ) |  |

A = A - B

Parameters
:   |  |  |
    | --- | --- |
    | B | another matrix |

Returns
:   A - B

|  |  |  |  |  |
| --- | --- | --- | --- | --- |
| double Jama.Matrix.norm1 | ( |  | ) |  |

One norm

Returns
:   maximum column sum.

|  |  |  |  |  |
| --- | --- | --- | --- | --- |
| double Jama.Matrix.norm2 | ( |  | ) |  |

Two norm

Returns
:   maximum singular value.

|  |  |  |  |  |
| --- | --- | --- | --- | --- |
| double Jama.Matrix.normF | ( |  | ) |  |

Frobenius norm

Returns
:   sqrt of sum of squares of all elements.

|  |  |  |  |  |
| --- | --- | --- | --- | --- |
| double Jama.Matrix.normInf | ( |  | ) |  |

Infinity norm

Returns
:   maximum row sum.

|  |  |  |  |  |  |
| --- | --- | --- | --- | --- | --- |
| Matrix Jama.Matrix.plus | ( | Matrix | *B* | ) |  |

C = A + B

Parameters
:   |  |  |
    | --- | --- |
    | B | another matrix |

Returns
:   A + B

Here is the call graph for this function:

|  |  |  |  |  |  |
| --- | --- | --- | --- | --- | --- |
| Matrix Jama.Matrix.plusEquals | ( | Matrix | *B* | ) |  |

A = A + B

Parameters
:   |  |  |
    | --- | --- |
    | B | another matrix |

Returns
:   A + B

|  |  |  |  |
| --- | --- | --- | --- |
| void Jama.Matrix.print | ( | int | *w*, |
|  |  | int | *d* |
|  | ) |  |  |

Print the matrix to stdout. Line the elements up in columns with a Fortran-like 'Fw.d' style format.

Parameters
:   |  |  |
    | --- | --- |
    | w | Column width. |
    | d | Number of digits after the decimal. |

Here is the caller graph for this function:

|  |  |  |  |
| --- | --- | --- | --- |
| void Jama.Matrix.print | ( | PrintWriter | *output*, |
|  |  | int | *w*, |
|  |  | int | *d* |
|  | ) |  |  |

Print the matrix to the output stream. Line the elements up in columns with a Fortran-like 'Fw.d' style format.

Parameters
:   |  |  |
    | --- | --- |
    | output | Output stream. |
    | w | Column width. |
    | d | Number of digits after the decimal. |

Here is the call graph for this function:

|  |  |  |  |
| --- | --- | --- | --- |
| void Jama.Matrix.print | ( | NumberFormat | *format*, |
|  |  | int | *width* |
|  | ) |  |  |

Print the matrix to stdout. Line the elements up in columns. Use the format object, and right justify within columns of width characters. Note that is the matrix is to be read back in, you probably will want to use a NumberFormat that is set to US Locale.

Parameters
:   |  |  |
    | --- | --- |
    | format | A Formatting object for individual elements. |
    | width | Field width for each column. |

See also
:   java.text.DecimalFormat::setDecimalFormatSymbols

Here is the call graph for this function:

|  |  |  |  |
| --- | --- | --- | --- |
| void Jama.Matrix.print | ( | PrintWriter | *output*, |
|  |  | NumberFormat | *format*, |
|  |  | int | *width* |
|  | ) |  |  |

Print the matrix to the output stream. Line the elements up in columns. Use the format object, and right justify within columns of width characters. Note that is the matrix is to be read back in, you probably will want to use a NumberFormat that is set to US Locale.

Parameters
:   |  |  |
    | --- | --- |
    | output | the output stream. |
    | format | A formatting object to format the matrix elements |
    | width | Column width. |

See also
:   java.text.DecimalFormat::setDecimalFormatSymbols

|  |  |  |  |  |
| --- | --- | --- | --- | --- |
| QRDecomposition Jama.Matrix.qr | ( |  | ) |  |

QR Decomposition

Returns
:   QRDecomposition

See also
:   QRDecomposition

|  |  |  |  |  |
| --- | --- | --- | --- | --- |
| void Jama.Matrix.quantileNormalizeColumns | ( |  | ) |  |

Here is the call graph for this function:

|  |  |  |  |
| --- | --- | --- | --- |
| static Matrix Jama.Matrix.random | ( | int | *m*, |
|  |  | int | *n* |
|  | ) |  |  |

static

Generate matrix with random elements

Parameters
:   |  |  |
    | --- | --- |
    | m | Number of rows. |
    | n | Number of colums. |

Returns
:   An m-by-n matrix with uniformly distributed random elements.

Here is the call graph for this function:

|  |  |  |  |  |
| --- | --- | --- | --- | --- |
| int Jama.Matrix.rank | ( |  | ) |  |

Matrix rank

Returns
:   effective numerical rank, obtained from SVD.

|  |  |  |  |  |  |
| --- | --- | --- | --- | --- | --- |
| static Matrix Jama.Matrix.read | ( | BufferedReader | *input* | ) | throws java.io.IOException |

static

Read a matrix from a stream. The format is the same the print method, so printed matrices can be read back in (provided they were printed using US Locale). Elements are separated by whitespace, all the elements for each row appear on a single line, the last row is followed by a blank line.

Parameters
:   |  |  |
    | --- | --- |
    | input | the input stream. |

Here is the call graph for this function:

|  |  |  |  |
| --- | --- | --- | --- |
| void Jama.Matrix.set | ( | int | *i*, |
|  |  | int | *j*, |
|  |  | double | *s* |
|  | ) |  |  |

Set a single element.

Parameters
:   |  |  |
    | --- | --- |
    | i | Row index. |
    | j | Column index. |
    | s | A(i,j). |

Exceptions
:   |  |  |
    | --- | --- |
    | ArrayIndexOutOfBoundsException |  |

Here is the caller graph for this function:

|  |  |  |  |
| --- | --- | --- | --- |
| void Jama.Matrix.setColumn | ( | int | *column*, |
|  |  | double[] | *vals* |
|  | ) |  |  |

Here is the call graph for this function:

|  |  |  |  |
| --- | --- | --- | --- |
| void Jama.Matrix.setMatrix | ( | int | *i0*, |
|  |  | int | *i1*, |
|  |  | int | *j0*, |
|  |  | int | *j1*, |
|  |  | Matrix | *X* |
|  | ) |  |  |

Set a submatrix.

Parameters
:   |  |  |
    | --- | --- |
    | i0 | Initial row index |
    | i1 | Final row index |
    | j0 | Initial column index |
    | j1 | Final column index |
    | X | A(i0:i1,j0:j1) |

Exceptions
:   |  |  |
    | --- | --- |
    | ArrayIndexOutOfBoundsException | Submatrix indices |

|  |  |  |  |
| --- | --- | --- | --- |
| void Jama.Matrix.setMatrix | ( | int[] | *r*, |
|  |  | int[] | *c*, |
|  |  | Matrix | *X* |
|  | ) |  |  |

Set a submatrix.

Parameters
:   |  |  |
    | --- | --- |
    | r | Array of row indices. |
    | c | Array of column indices. |
    | X | A(r(:),c(:)) |

Exceptions
:   |  |  |
    | --- | --- |
    | ArrayIndexOutOfBoundsException | Submatrix indices |

|  |  |  |  |
| --- | --- | --- | --- |
| void Jama.Matrix.setMatrix | ( | int[] | *r*, |
|  |  | int | *j0*, |
|  |  | int | *j1*, |
|  |  | Matrix | *X* |
|  | ) |  |  |

Set a submatrix.

Parameters
:   |  |  |
    | --- | --- |
    | r | Array of row indices. |
    | j0 | Initial column index |
    | j1 | Final column index |
    | X | A(r(:),j0:j1) |

Exceptions
:   |  |  |
    | --- | --- |
    | ArrayIndexOutOfBoundsException | Submatrix indices |

|  |  |  |  |
| --- | --- | --- | --- |
| void Jama.Matrix.setMatrix | ( | int | *i0*, |
|  |  | int | *i1*, |
|  |  | int[] | *c*, |
|  |  | Matrix | *X* |
|  | ) |  |  |

Set a submatrix.

Parameters
:   |  |  |
    | --- | --- |
    | i0 | Initial row index |
    | i1 | Final row index |
    | c | Array of column indices. |
    | X | A(i0:i1,c(:)) |

Exceptions
:   |  |  |
    | --- | --- |
    | ArrayIndexOutOfBoundsException | Submatrix indices |

|  |  |  |  |
| --- | --- | --- | --- |
| boolean Jama.Matrix.setRow | ( | int | *row*, |
|  |  | double[] | *vals* |
|  | ) |  |  |

Here is the call graph for this function:

|  |  |  |  |
| --- | --- | --- | --- |
| boolean Jama.Matrix.setRow | ( | int | *row*, |
|  |  | int[] | *vals* |
|  | ) |  |  |

Here is the call graph for this function:

|  |  |  |  |  |  |
| --- | --- | --- | --- | --- | --- |
| Matrix Jama.Matrix.solve | ( | Matrix | *B* | ) |  |

Solve A\*X = B

Parameters
:   |  |  |
    | --- | --- |
    | B | right hand side |

Returns
:   solution if A is square, least squares solution otherwise

Here is the caller graph for this function:

|  |  |  |  |  |  |
| --- | --- | --- | --- | --- | --- |
| Matrix Jama.Matrix.solveTranspose | ( | Matrix | *B* | ) |  |

Solve X\*A = B, which is also A'\*X' = B'

Parameters
:   |  |  |
    | --- | --- |
    | B | right hand side |

Returns
:   solution if A is square, least squares solution otherwise.

Here is the call graph for this function:

|  |  |  |  |  |
| --- | --- | --- | --- | --- |
| Matrix Jama.Matrix.sortColumns | ( |  | ) |  |

Here is the call graph for this function:

|  |  |  |  |  |  |
| --- | --- | --- | --- | --- | --- |
| Matrix Jama.Matrix.sortColumnsKeepingIndex | ( | Matrix | *index* | ) |  |

Here is the call graph for this function:

Here is the caller graph for this function:

|  |  |  |  |  |
| --- | --- | --- | --- | --- |
| SingularValueDecomposition Jama.Matrix.svd | ( |  | ) |  |

Singular Value Decomposition

Returns
:   SingularValueDecomposition

See also
:   SingularValueDecomposition

|  |  |  |  |  |  |
| --- | --- | --- | --- | --- | --- |
| Matrix Jama.Matrix.times | ( | double | *s* | ) |  |

Multiply a matrix by a scalar, C = s\*A

Parameters
:   |  |  |
    | --- | --- |
    | s | scalar |

Returns
:   s\*A

Here is the call graph for this function:

|  |  |  |  |  |  |
| --- | --- | --- | --- | --- | --- |
| Matrix Jama.Matrix.times | ( | Matrix | *B* | ) |  |

Linear algebraic matrix multiplication, A \* B

Parameters
:   |  |  |
    | --- | --- |
    | B | another matrix |

Returns
:   Matrix product, A \* B

Exceptions
:   |  |  |
    | --- | --- |
    | IllegalArgumentException | Matrix inner dimensions must agree. |

Here is the call graph for this function:

|  |  |  |  |  |  |
| --- | --- | --- | --- | --- | --- |
| Matrix Jama.Matrix.timesEquals | ( | double | *s* | ) |  |

Multiply a matrix by a scalar in place, A = s\*A

Parameters
:   |  |  |
    | --- | --- |
    | s | scalar |

Returns
:   replace A by s\*A

|  |  |  |  |  |
| --- | --- | --- | --- | --- |
| String Jama.Matrix.toString | ( |  | ) |  |

|  |  |  |  |  |
| --- | --- | --- | --- | --- |
| double Jama.Matrix.trace | ( |  | ) |  |

Matrix trace.

Returns
:   sum of the diagonal elements.

|  |  |  |  |  |
| --- | --- | --- | --- | --- |
| Matrix Jama.Matrix.transpose | ( |  | ) |  |

Matrix transpose.

Returns
:   A'

Here is the call graph for this function:

Here is the caller graph for this function:

|  |  |  |  |  |
| --- | --- | --- | --- | --- |
| Matrix Jama.Matrix.uminus | ( |  | ) |  |

Unary minus

Returns
:   -A

Here is the call graph for this function:

|  |  |  |  |
| --- | --- | --- | --- |
| void Jama.Matrix.write | ( | String | *save*, |
|  |  | double[] | *observed* |
|  | ) |  | throws IOException |

---

The documentation for this class was generated from the following file:

- java/Jama/Matrix.java


---

Generated on Fri Aug 15 2014 15:11:06 for ESAT by  

 1.8.7
